# Assortative mating and fragmentation within dog breeds

**DOI:** 10.1186/1471-2148-8-28

**Published:** 2008-01-28

**Authors:** Susanne Björnerfeldt, Frank Hailer, Maria Nord, Carles Vilà

**Affiliations:** 1 Department of Evolutionary Biology, Uppsala University, S-752 36 Uppsala, Sweden; 2 Department of Animal Breeding and Genetics, Section of Molecular Animal Genetics, BMC, Box 597, S-751 24Uppsala, Sweden; 3 Center for Conservation and Evolutionary Genetics, National Zoological Park, National Museum of Natural History, Smithsonian Institution, 3001 Connecticut Ave., NW, Washington, DC 20008, USA

## Abstract

**Background:**

There are around 400 internationally recognized dog breeds in the world today, with a remarkable diversity in size, shape, color and behavior. Breeds are considered to be uniform groups with similar physical characteristics, shaped by selection rooted in human preferences. This has led to a large genetic difference between breeds and a large extent of linkage disequilibrium within breeds. These characteristics are important for association mapping of candidate genes for diseases and therefore make dogs ideal models for gene mapping of human disorders. However, genetic uniformity within breeds may not always be the case. We studied patterns of genetic diversity within 164 poodles and compared it to 133 dogs from eight other breeds.

**Results:**

Our analyses revealed strong population structure within poodles, with differences among some poodle groups as pronounced as those among other well-recognized breeds. Pedigree analysis going three generations back in time confirmed that subgroups within poodles result from assortative mating imposed by breed standards as well as breeder preferences. Matings have not taken place at random or within traditionally identified size classes in poodles. Instead, a novel set of five poodle groups was identified, defined by combinations of size and color, which is not officially recognized by the kennel clubs. Patterns of genetic diversity in other breeds suggest that assortative mating leading to fragmentation may be a common feature within many dog breeds.

**Conclusion:**

The genetic structure observed in poodles is the result of local mating patterns, implying that breed fragmentation may be different in different countries. Such pronounced structuring within dog breeds can increase the power of association mapping studies, but also represents a serious problem if ignored.

In dog breeding, individuals are selected on the basis of morphology, behaviour, working or show purposes, as well as geographic population structure. The same processes which have historically created dog breeds are still ongoing, and create further subdivision within current dog breeds.

## Background

The behavioral and morphological diversity present in modern purebred dogs is remarkable. For at least 4,000–5,000 years, dogs have existed in a variety of sizes and shapes, but the majority of the currently recognized 400 breeds have only existed since around 1850 or later, when dog shows became popular, Kennel Clubs were founded and Stud Books were established [1,2]. In parallel to phenotypic selection, genetic differentiation between breeds has increased and is today very large [3-5]. The reasons for this high degree of genetic differentiation are the reproductive isolation among breeds, founder effects and bottlenecks experienced during the time of breed creation or later, extreme selection and use of popular sires [3,6-11].

The isolation and small effective population size of modern breeds has resulted in widespread inbreeding and the expression of a large number of genetic diseases. Many of these are also among the most frequently occurring diseases in humans, such as cancer, heart problems, deafness, blindness and joint diseases [6,8,9,12-14]. The genetic disorders in the dog often mimic human diseases closely in physiology, disease presentation and clinical response. More than 360 genetic diseases that are found in humans have also been described in dogs, about 46% occurring mainly in either one or a few breeds [8,10,15]. The fact that purebred dogs are separated into genetically differentiated breeds and that they live in the same environment as humans, makes them an ideal model to map genes for human disorders [5,8,13]. Another advantage is that linkage disequilibrium (LD) in dog breeds extends over 20–100 times longer genomic regions than in humans, which means that a smaller number of markers are required for genome-wide scans in dogs. This, together with the low haplotype diversity within LD regions and high degree of haplotype sharing among breeds [9,10,14,16], facilitates the identification of chromosomal regions where candidate genes are located.

Association mapping of phenotypic traits or diseases is a population-based approach (instead of pedigree-based) and depends highly on the level of LD [14], which in dogs is related to the genetic uniformity of breeds. Dogs are today divided into different breeds based mainly on morphological appearance and/or behavior. Breeds are defined as "intraspecies groups that have relatively uniform physical characteristics developed under controlled conditions by man [4]." However, homogeneity within breeds may not always be the case. For example, the official standards for many breeds allow a number of color types and patterns, while other combinations are unacceptable [17]. Variation in size can lead to the identification of different types within a breed. Additionally, breeders are increasingly interested in specializing in either dogs for show, or dogs for working purposes or as pets. These divergent selective forces within breeds are a form of disruptive selection: extreme forms are favored while intermediate forms are selected against [18]. Since human preferences play the same role of assortative mating in keeping the breeding lines separate, these are likely to lead to the formation of genetically separated groups, thus violating the assumption of uniformity within breeds.

One example of this breed fragmentation is the case of the poodle. According to the World Canine Organization, FCI (Fédération Cynologique Internationale) [19], poodles are grouped in four different sizes: standard (45–60 cm, withers height), medium sized (35–45 cm), miniature (28–35 cm) and toy (< 28 cm). At the same time, five uniform colors are allowed: black, brown, white, silver and apricot [19]. Despite the different sizes and colors, all poodles are currently recognized as a single breed. Disruptive selection for very specific phenotypes and assortative mating might have led to fragmentation, and to genetically differentiated types within this breed. In this study we used purebred poodles from Sweden as an example to study how genetic diversity at 27 microsatellite loci is partitioned within a single dog breed, and then compare this pattern to variation within and among eight other purebred dog breeds. This knowledge can contribute to a better understanding of the history and relationship between breeds, exemplifies the genetic diversification of populations as a result of disruptive selection, and it is also vital to the design of optimal search strategies for association mapping of diseases or phenotypic traits [9,20].

## Results

The study of 164 Swedish poodles revealed an average of 4.4 alleles per locus, a significant (p < 0.05) heterozygote deficit across all 27 markers (H_E _= 0.64, H_O _= 0.56), and positive inbreeding coefficient (F_IS _= 0.133). This heterozygote deficit was consistently found in 20 of 27 markers, indicating that the deviation from Hardy-Weinberg equilibrium affects the whole genome rather than being a local, locus-specific phenomenon such as selection or genotyping problems (e.g. allelic dropout). Heterozygote deficit could indicate a preferential mating with relatives within poodles, or the presence of population structure within the breed (Wahlund effect) [21].

To assess whether the heterozygote deficit was due to the fragmentation of poodles into the four discrete size classes acknowledged by FCI, we measured the degree of population differentiation using F_ST _[22] (Table 1). Standard poodles were very well separated from the smaller poodles, with pairwise F_ST _values in the range of 0.184 to 0.234 (p < 0.01 in all cases), which suggests very limited genetic exchange between them. On the other hand, the differentiation between the three smaller size classes (medium sized, miniature and toy) is much lower, with F_ST _values at least 6.8 times smaller than the smallest value observed between any of them and standard poodles. Among them, miniature and toy poodles were indistinguishable (F_ST _not significantly different from 0) (Table 1).

**Table 1:** Genetic differentiation between four size classes of poodles.

F_ST_	Medium sized poodle	Miniature poodle	Toy poodle
Standard poodle	0.234**	0.195**	0.184**
Medium sized poodle		0.014	0.027**
Miniature poodle			-0.001

Measured as pairwise F_ST _(Weir and Cockerham 1984), based on variation at 27 microsatellite loci. *p < 0.05; **p < 0.01; ***p < 0.001.

**Figure 1:**
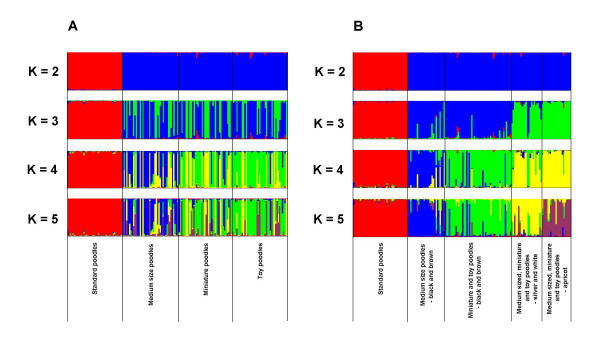
**Clustering assignment of 164 purebred poodles**. Results from STRUCTURE 2.1. Each individual is represented as a bar, divided into K colors, where K is the number of clusters assumed (K = 2–5). (A) Individuals are sorted according to the four sizes recognized in poodles. (B) Individuals are sorted according to color and size combinations.

We used the program STRUCTURE to identify the groups into which poodles are divided. We determined the number of existing clusters in two ways, by considering the log 'probability of data' *lnP(D) *for different numbers of K, and by using the statistic ΔK [23], which considers the rate of change in *lnP(D) *among successive K values. The latter approach indicated that the optimal number of groups was K = 2. However, *lnP(D) *continued to increase with increasing K until K = 5, and thereafter *lnP(D) *decreased. At K = 2, standard poodles appeared separated from the smaller poodles (medium sized, miniature and toy), and they remained separated with increasing K values. Larger K values failed to separate the other sizes (Figure 1A).

**Figure 2:**
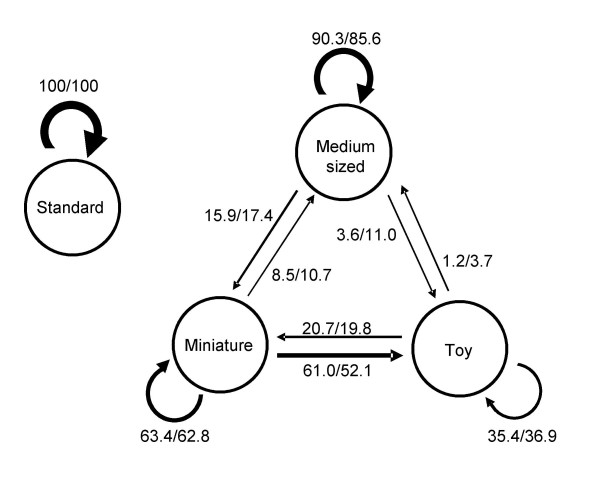
**Proportion (%) of ancestry contribution for poodle groups based on pedigree information**. Thickness of arrows indicates percentage of contribution from parents (left number) and great-grandparents (right number) to the dogs in each size class.

To corroborate that the genetic differentiation observed between the poodle size classes is inversely related to the current gene flow between them, we examined pedigree information from the Swedish Kennel Club [24]. We recorded the size registered for the parents, grandparents and great-grandparents for all 164 poodles studied. The pedigree information indicated that the gene flow estimates between the different poodle size classes are almost identical considering parents, grandparents (data not shown) or great-grandparents, suggesting that the patterns of gene exchange have remained relatively constant over recent times (Figure 2). The pedigree information confirms the complete isolation of standard poodles (100% of the parents, grandparents and great-grandparents were standard poodles). Similarly, about 87% of the ancestors of the medium sized poodles belonged to the medium size size-class, while the corresponding proportions were only around 63% for miniature and around 36% for toy. The genetic exchange between the two smallest sizes thus appeared to be very large and 52–61% of the ancestors (based on the number of parents and great-grandparents, Figure 2) of toy were in fact miniature poodles. The patterns of genetic exchange suggested by the pedigree closely parallel the genetic differentiation between the size classes (Table 1), as confirmed by a Mantel correlation test (Z = 0.650, p = 0.039).

Since the analysis with STRUCTURE suggested that the likelihood of the clustering within poodles was highest with K = 5, we examined more closely the characteristics for each dog available at the registry. This revealed that the five groups at K = 5 could be clearly defined by a combination of size and coat-color. Multiple runs of the program consistently provided the same results. After rearranging the results of STRUCTURE shown in Figure 1A according to size and color criteria, the division between groups could be seen more clearly (Figure 1B). As indicated above, at K = 2, the first group to separate from the rest corresponds to the standard poodles. At K = 3, a new group is formed within the smaller size classes of poodles by separating individuals with black and brown coat colors from those with the other three colors (silver, white and apricot). At K = 4 the group of black and brown small sized poodles is further subdivided and medium sized form a separate group from miniature and toy. Finally, at K = 5 the group containing the small (medium sized, miniature and toy) poodles of pale colors, is separated into two groups: one containing silver (grey) and white dogs and the other dogs with apricot color. Consequently, our results indicate that the genetic diversity within poodles is clearly separated into two groups which differ by size (standard versus smaller poodles) and that actually five separate clusters can be discerned based on a combination of color and size. Using F_ST _to measure the degree of differentiation between the five groups (Table 2) confirms that standard poodles are very different from the three smaller-bodied groups of poodles (pairwise F_ST _= 0.197 to 0.265). However, the differentiation among the four groups of smaller poodles, defined on the basis of color and size, is about four times larger than the largest difference observed between the size classes for the three small-sized poodles, reaching F_ST _values of up to 0.120 (Table 1).

**Table 2:** Genetic differentiation among poodle groups and other dogs.

F_ST_	MS brown black	M, T brown black	MS, M, T silver white	MS, M, T apricot	Miniature schnauzer	Giant schna.	Fox terrier (smooth)	Fox terrier (wire)	Bull terrier	German shep.	Labrador retriever	Siberian husky
Standard poodle	0.265	0.199	0.197	0.262	0.279	0.273	0.372	0.346	0.367	0.332	0.278	0.241
MS brown-black^1^		0.050	0.085	0.120	0.255	0.214	0.293	0.301	0.368	0.252	0.214	0.207
M, T brown-black^2^			0.052	0.110	0.219	0.188	0.238	0.238	0.309	0.248	0.157	0.182
MS, M, T silver-white^3^				0.059	0.179	0.170	0.220	0.225	0.274	0.232	0.117	0.150
MS, M, T apricot^4^					0.248	0.226	0.275	0.304	0.327	0.306	0.197	0.235
Miniature schnauzer						0.150	0.321	0.332	0.402	0.301	0.191	0.194
Giant schnauzer							0.292	0.318	0.386	0.195	0.212	0.172
Fox terrier (smooth)								0.285	0.444	0.414	0.290	0.290
Fox terrier (wire)									0.452	0.424	0.272	0.270
Bull terrier										0.517	0.306	0.371
German shepherd											0.273	0.235
Labrador retriever												0.187

Measured as pairwise F_ST _(Weir and Cockerham 1984), between five size and color groups found in poodles (within box, see text) and eight additional breeds, for 27 loci. All results are highly significant (p < 0.01).

^1 ^Medium size poodles with brown or black color

^2 ^Miniature poodles and toy poodles with brown or black color

^3 ^Medium size poodles, miniature poodles and toy poodles with silver (grey) or white color

^4 ^Medium size poodles, miniature poodles and toy poodles with apricot color

A reanalysis of the pedigree information revealed that the clusters shown by the STRUCTURE analysis matched better the breeding practices for poodles. For each one of the five groups defined, more than 80% of the parents and great-grandparents originated from the same group (data not shown), as compared to the much lower values for the size classes (just 35% for toy poodles, Figure 2). Our analysis of pedigree and phenotype data thus confirmed that the genetic clusters identified within poodles are the result of specific breeding preferences of dog owners, and not just originating from mating related dogs. To our knowledge, these groups have not been identified or suggested before, thus our genetic analysis revealed hidden population substructure within poodles.

**Figure 3:**
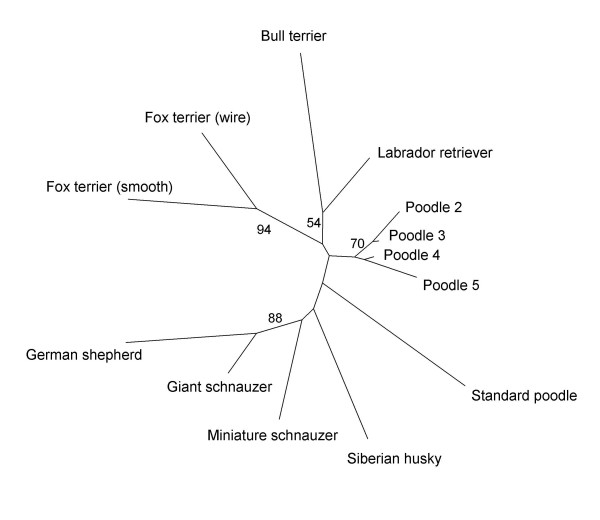
**Genetic similarity between poodle subgroups and eight other dog breeds**. Neighbor-joining analysis of allelic composition at 27 microsatellite loci. Calculations are based on Nei's [25] standard genetic distances (D_S_), and numbers on branches correspond to bootstrap values from 1000 bootstrap resamplings across loci (only values above 50 are shown). Poodle 2, black and brown medium sized poodles; Poodle 3, black and brown miniature and toy poodles; Poodle 4, silver and white medium sized, miniature and toy poodles; Poodle 5, apricot medium sized, miniature and toy poodles.

These five groups of poodles were also genetically compared to 133 dogs from eight other breeds (Table 2). Standard poodles were as differentiated from smaller poodles as different breeds are from each other (pairwise F_ST _= 0.150–0.517). For instance, the pairwise F_ST _value between standard poodles and black and brown medium sized poodles was 0.265, larger than 40% of the comparisons between the five groups of poodles and the other 8 breeds included in this study. For example, the F_ST _value between giant schnauzer and small white and silver poodles is just 0.170. A neighbor-joining analysis (Figure 3) based on another measure of genetic differentiation, Nei's [25] standard genetic distance (D_S_), shows standard poodles at the end of a long branch, indicating genetic uniqueness and separation from all other poodles.

One possible explanation for the large difference between standard and small sized poodles could be a past bottleneck that reduced the diversity in the first compared to the latter. This bottleneck could translate into a strong differentiation by founder effects and drift. However, this does not seem to be the main reason for their uniqueness, because their allelic richness was similar to that observed in other groups of poodles after correcting for differences in sample size (Table 3).

**Table 3:** Genetic variation within poodle groups and other dog breeds.

Population	Sample size	Sample size/locus	F_IS_	Unbiased H_E _(SD)	H_O _(SD)	Bootstrapped allelic richness
All poodles	164	164	0.133	0.641 ± 0.027	0.556 ± 0.008***	4.4
Standard poodle	41	41	-0.008	0.533 ± 0.032	0.538 ± 0.015	3.6
MS, M, T poodles^1^	123	123	0.072	0.605 ± 0.029	0.562 ± 0.009***	4.1
MS brown-black^2^	28	28	-0.001	0.526 ± 0.039	0.527 ± 0.018	3.6
M, T brown-black^3^	50	50	-0.013	0.583 ± 0.031	0.591 ± 0.013	3.8
MS, M, T silver-white^4^	23	23	0.023	0.634 ± 0.024	0.620 ± 0.020	4.0
MS, M, T apricot^5^	22	22	0.107	0.536 ± 0.034	0.480 ± 0.021***	3.5
Miniature schnauzer	14	10.2	0.074	0.520 ± 0.036	0.483 ± 0.030*	(3.0)^6^
Giant schnauzer	17	13.7	0.122	0.568 ± 0.039	0.501 ± 0.026***	3.5
Fox terrier (smooth)	18	14.2	0.029	0.456 ± 0.042	0.443 ± 0.025	2.8
Fox terrier (wire)	18	15.0	0.100	0.419 ± 0.044	0.380 ± 0.024**	2.6
Bull terrier	18	15.3	0.090	0.314 ± 0.047	0.287 ± 0.022	1.9
German shepherd	16	11.0	0.135	0.447 ± 0.043	0.394 ± 0.028*	2.6
Labrador retriever	16	12.7	0.042	0.563 ± 0.036	0.543 ± 0.027*	3.3
Siberian husky	16	13.3	0.078	0.659 ± 0.035	0.611 ± 0.026***	4.1

Number of dogs tested, average sample size per locus, inbreeding coefficient (F_IS_), expected and observed heterozygosity (H_E _and H_O_; SD, standard deviation), and allelic richness corrected for sample size (mean number of alleles per locus when 14 individuals – the sample size for miniature schnauzer; sampled with replacement). Significance refers to the difference between H_E _and H_O_, *p < 0.05; **p < 0.01; ***p < 0.001.

^1 ^Medium sized poodles, miniature poodles and toy poodles

^2 ^Medium sized poodles with brown or black coat-color

^3 ^Miniature poodles and toy poodles with brown or black coat-color

^4 ^Medium sized poodles, miniature poodles and toy poodles with silver (grey) or white coat-color

^5 ^Medium sized poodles, miniature poodles and toy poodles with apricot coat-color

^6 ^Without size correction

In order to compare the uniqueness of the five poodle groups in relation to the differentiation between recognized dog breeds we conducted assignment tests. If breeds (and groups within poodles) are well differentiated, most of the individuals should be correctly assigned to their nominal group. In a first test we divided all poodles into two groups (standard poodles and smaller sizes, as suggested by STRUCTURE for K = 2), and compared them to dogs from 8 other breeds. Only 2 out of 297 dogs were incorrectly assigned and none of these mis-assignments corresponded to poodles. This indicates that established dog breeds are well-isolated populations with unique microsatellite allele frequencies (as previously shown by Refs. [1,3,4]), and that a similar degree of differentiation exists between standard poodles and the group of small sized poodles. A second assignment test was made examining only the four coat-color/size groups of small poodles. This resulted in a high self-assignment, but 20 out of 123 were matched to a different color/size combination, showing a lower degree of differentiation between groups within small poodles.

Poodles could represent a unique case among dogs if they developed from the mixing of separate dog lineages. If this was the case, we would expect to find a larger genetic diversity in poodles than that observed in other breeds and, more importantly, a larger inbreeding coefficient F_IS _due to population fragmentation (Wahlund effect; [21]). When considering all poodles as members of a single group, they show high genetic diversity (H_E_, Table 3) and a large inbreeding coefficient F_IS_, further evidenced by a significant heterozygote deficit. However, several other dog breeds also had high levels of microsatellite diversity H_E _(i.e., Siberian husky), F_IS _values as large as those in poodles (i.e., German shepherd) or significant heterozygote deficits (6 different breeds, see Table 3). Although the allelic richness corrected for sample size was larger when all poodles were considered as one single group, the differences compared to the other breeds were small (Table 3). It could be argued that differences in genetic diversity between breeds are the result of differences in population size. However, this does not seem to be the case. Genetic diversity (bootstrapped allelic richness, Table 3) was not correlated to breed population size as inferred by the number of registries at the Swedish Kennel Club during 2005 (r = 0.207, p = 0.592, data not shown). Similarly, the inbreeding coefficient F_IS _was not correlated with breed population size either (r = 0.217, p = 0.576). Since genetic diversity and inbreeding coefficient are not related to population size, the large differences in diversity and positive inbreeding coefficient in some breeds could be indicative of some degree of genetic fragmentation within these breeds, similar to that observed in poodles.

## Discussion

About 400 dog breeds exist today. All of these breeds are characterized by unique morphology, characteristic behavior and often also by a suite of genetic diseases. The morphological differences between breeds are so large that they easily exceed the differentiation between all species in the Family Canidae [26,27], and probably no other vertebrate has comparable phenotypic diversity. Our results suggest that currently the number of genetically differentiated groups of purebred dogs might be even larger than the number of breeds: some breeds, such as the poodle, are likely to encompass multiple genetically divergent subgroups. Selection patterns within breeds might lead to an increase in the number of genetically differentiated dog breeds.

Poodles represent a breed encompassing apparent phenotypic diversity, which translates in the identification of four classes with regard to body size. However, our analyses revealed that this is not the only reason for the poodle intra-breed structure. Only a limited set of coat colors are accepted in purebred poodles, as for most modern breeds, and coats are required to be uniform [17,19]. Since other colors and coat patterns are not acceptable in registered purebred poodles, this results in disruptive selection by which some extreme combinations are favored while intermediate types are rejected. As a consequence of this, the genetic diversity of poodles is fragmented into five distinct groups defined by a combination of size and color (Figure 1B). One of the groups, standard poodles, appears as different from the other poodle groups as recognized dog breeds are different from each other (Table 2). This result emphasizes how strong the intra-breed differentiation can be.

Historically, standard poodles were used as working dogs, while the three smaller poodle classes were mostly bred for company. Standard poodles have been bred separately from the smaller sizes for more than 100 years in Europe (Barbro Teglöf, responsible for the breeding committee of the Swedish Poodle Club, personal communication). In the US, however, some degree of interbreeding among the recognized poodle size classes occurs. This could result in different patterns of differentiation in American poodles. Our results show a strong differentiation between standard poodles and the smaller sizes (Figure 3, Table 2). Due to these different breeding practices, it is possible that standard poodles are not as differentiated from small sized poodles in the US as they are in Europe. This study is based on poodles born in Sweden; population structure in other countries may be similarly affected by local regulations and breeding practices.

The long isolation between standard poodles and the smaller sizes in Europe has resulted in a differentiation as pronounced as that between well defined breeds. Founder effects and subsequent bottlenecks during the history of breeds, together with highly selective breeding practices [9,11], have led to the differentiation between breeds and probably have also led to the strong separation between standard poodles and the group of small sized poodles. This process results in a rapid random genetic drift and unique allele frequencies. Consequently, attempts to reconstruct the relationship between breeds based on their allelic composition [3-5,28,29] are likely to have been heavily influenced by such random effects (Figure 3) and may not necessarily reflect true breed history. For example, our results suggest that, for neutral genetic markers, standard poodles are as differentiated from black and brown medium sized poodles as from giant schnauzers or Siberian huskies (Table 2). This indicates that genetic distance based on neutral allelic frequencies might actually be a poor indicator of breed relatedness.

The four groups within the small sized poodles show lower levels of differentiation, below the values commonly observed between the other breeds or between them and standard poodles (Figure 1B, Table 2). Nevertheless, our results confirm that these dogs do not constitute a panmictic breeding population where all individuals randomly breed to each other. The situation observed in this study for the poodles, intra-breed structure, may be present in other breeds as well, as suggested by the large inbreeding coefficient in some of the breeds (Table 3), consistent with population fragmentation [30]. Similarly, Schelling, Gaillard and Dolf [29] observed that while longhaired and smooth dachshunds were genetically similar, wirehaired dachshunds clustered separately in a phylogenetic tree. For some breeds such as German shepherds, Siberian huskies and Labrador retrievers, two different lines of selection are maintained within the breed, separating animals intended for competition in dog shows for their appearance, and animals selected for work. In these cases the morphology of the two types is becoming more and more divergent over time, which will likely result in an intra-breed population structure comparable to that observed in poodles. The existence of separated lines of selective breeding within the same breed is likely to lead to the establishment of distinct genetic clusters within recognized dog breeds. Thus, an ongoing process of selective breeding is leading to a progressive increase in the number of dog breeds, and this increase will continue as long as the popularity of purebred dogs and dog shows continues.

## Conclusion

The partitioning of genetic diversity into discrete classes (breeds) has made the dog an exceptional model for the study of the association between genotype and phenotype and for the identification of genes involved in phenotypic and behavioral traits or diseases [8,10,14,15,20]. However, the use of dogs as a model is determined by the uniformity within breeds. Our results suggest that genetically divergent groups of dogs can exist within the same breed. The implications of this for association mapping studies are two-fold. First, the number of genetically identifiable breeds may be even larger than suspected (about 400), increasing the value of dogs as a model organism for association studies. Since these genetically differentiated groups are likely to include only a small part of the haplotype diversity attributed to the breed and to represent more inbred lines, LD is expected to be larger [14]. This would facilitate the identification of markers linked to the trait under study. Second, this intra-breed structure violates the assumption of uniformity within breeds. Such cryptic (unrecognized by the official kennel clubs' policies) population structure, if ignored, is likely to confound association studies. Association studies should target genetically homogeneous groups within breeds, which may often be phenotypically and/or behaviorally separated.

Assortative mating is leading to the creation of strong population structure within dog breeds and has contributed to the extreme plasticity of dogs and domestic animals under selection. Domestic animals are invaluable models to understand evolution, and in the same way that they were a major source of inspiration for Darwin [31], they continue to help us understand the origin of biodiversity.

## Methods

### Samples

Buccal swabs were taken from 297 purebred dogs registered by the Swedish Kennel Club (SKK) [24]. Of these samples, 164 corresponded to poodles (41 of each size: standard, medium sized, miniature and toy). The sampling also included bull terriers (n = 18), fox terriers (smooth) (n = 18), fox terriers (wire) (n = 18), German shepherds (n = 16), giant schnauzers (n = 17), miniature schnauzers (n = 14), Labrador retrievers (n = 16) and Siberian huskies (n = 16). Samples were collected at a dog show in December 2004, and by direct correspondence with dog owners during 2005. The registration numbers in the Swedish Kennel Club were recorded for all individuals to avoid sampling dogs that share any parent. For the poodles the registration numbers were also used to track the size and color of the ancestors (parents, grandparents and great-grandparents, although, to simplify, we do not show data for grandparents in this paper). The buccal cells were taken using nylon bristle cytology brushes (Medical Packaging Corp, Camarillo, CA) by brushing the inside of the dog's cheek for at least 20 seconds. The brush with the sample was immediately put into a tube with 1 ml Laird's buffer (0.1 M Tris-HCl, 5 mM EDTA, 0.2 M NaCl, 7 mM SDS, adjusted to pH 8.5). As soon as the samples arrived at the laboratory they were kept at -20°C until processing.

### Laboratory methods

Genomic DNA was extracted from 400 μl of the buffer containing the cytology brush with the sample, by digestion with 0.3 mg of proteinase K. The samples were then incubated over-night at 37°C and DNA was extracted using a modified phenol/chloroform protocol [32].

Twenty-eight biparentally inherited autosomal microsatellites, distributed across the canine genome, were typed for all dogs: Ren94K11 (mapped to canine chromosome CFA12), C17.402 (CFA17), Ren239K24 (CFA29), C18.460 (CFA18), Ren274F18 (CFA19), Ren181K04 (CFA23), C11.873 (CFA11), Ren73F08 (CFA10), Ren112I02 (CFA01), C02.894 (CFA02), Ren204K13 (CFA08), Ren160J02 (CFA04), Ren106I06 (CFA24) [33], FH3109 (CFA20), FH2887 (CFA20), FH2914 (CFA21), FH2785 (CFA28), FH2759 (CFA28) [34], Ren37H09 (CFA06), Ren49F22 (CFA22) [35], c2017 (CFA15) [36], u109 (CFA04), u225 (CFA10), u250 (CFA09) and u253 (CFA20) [37], vWF (CFA27) [38] and PEZ05 (CFA12) and PEZ12 (CFA03) [39]. The microsatellites were amplified by polymerase chain reaction (PCR). The amplifications for each sample were done in 14 reactions of 10 μl each, which included 12 multiplexes of two or three loci, and two loci amplified separately (PEZ05 and PEZ12). The PCR mix included 1x HotStar buffer (QIAGEN, Hilden, Germany), 0.25 mM dNTP, 0.32 μM of each primer, 3.0 mM MgCl_2_, 0.025x Q solution, 0.45U HotStarTaq and 2 μl DNA template. The PCR profile included an initial denaturation step at 95°C for 15 minutes followed by 10 touchdown cycles (30 s of denaturation at 95°C, 30 s annealing starting at 58°C and decreasing 0.5°C each cycle, followed by extension at 72°C for 45 s), followed by 20 additional cycles (denaturation at 95°C for 30 s, annealing at 53°C for 30 s and extension at 72°C for 45 s), and a final extension step at 72°C for 10 min in a PTC-0225 DNA Engine Tetrad (Bio-Rad). PCR products were pooled in seven different pools and genotyped on a MegaBACE 1000™ instrument (Amersham Biosciences). Genotypes were identified using the software Genetic Profiler v2.2 (Amersham Biosciences).

### Data analysis

For each microsatellite marker and breed, a test for deviation from Hardy-Weinberg equilibrium frequencies was performed using GENEPOP on the Web 3.1c [40]. A locus-by-locus analysis of deviations from Hardy-Weinberg equilibrium frequencies indicated that one marker (Ren181K04) showed a heterozygote deficiency for all breeds and groups of poodles, suggesting presence of null alleles, genotyping errors or tight physical linkage to a selected trait. This marker was excluded from all analyses.

We used the program Microsatellite Toolkit 3.1 [41] to calculate expected and observed heterozygosity (H_E _and H_O_) [42] for each breed and groups within poodles. Since sample size was different for each breed, we also used a bootstrapping procedure (as in Hailer et al.) [43] to compare levels of genetic diversity after correcting for sample size: 14 individuals (corresponding to the sample size for miniature schnauzer, for which the number of individuals studied was smallest) were randomly sampled 100 times with replacement from each original population sample, and the average number of alleles per locus was calculated across these 100 replicates. F_IS _values for each locus and across loci were calculated using FSTAT 2.9.3.2 [44].

We quantified the degree of differentiation between breeds in GENETIX 4.05 [45] by calculating pairwise F_ST _values [22]. Significance was assessed from 1000 permutations. Relationships between dog breeds were investigated by constructing a neighbor-joining (NJ) tree using Nei's [25] standard genetic distances (D_S_) with the program POPULATIONS [46]. Statistical support for the internal nodes of this tree was evaluated with 1000 bootstrap pseudoreplicates across loci. For poodles, F_ST _values were compared to gene flow estimates based on three generations of pedigree information using a Mantel test [47]. The pedigree-based matrix was calculated as 1-(average gene flow between groups); where the average gene flow between groups had been estimated from the great-grandparents of each individual dog (see Results, Figure 2); values of zero were assigned to the diagonal.

An additional estimate of the degree of differentiation between breeds (or between groups of individuals within poodles) was the proportion of self-assignment. The assignment program Doh [48] was used to evaluate if the individual genotypes allow correct assignment of each individual dog to its breed. The program uses the individual's genotype and determines the likelihood of finding that genotype within each breed. The individual is assigned to the breed for which it has the highest probability.

All the breed comparisons listed above involve groups of individuals defined *a priori *as belonging to one breed or another. We used the software STRUCTURE 2.1 [49,50] without population information, to form groups of poodles solely based on their genetic composition. This approach allowed us to identify how the genetic diversity is partitioned within poodles. We subdivided these dogs into an increasing number of populations (K = 1–10 with a burn-in length of 100,000 and a run length of 1,000,000) and performed five independent runs per K value to ensure that the results were consistent. The program was run allowing animals to have mixed ancestry and correlated allele frequencies. We compared the likelihood estimate for each one of the K values essayed in order to decide the number of subpopulations present within poodles. In subsequent comparisons we divided the poodles in the groups suggested by the program STRUCTURE and each group was treated as a separate subgroup and subjected to the analyses described above.

## Authors' contributions

CV designed and coordinated the study. SB and MN carried out all laboratory work. SB, FH and CV performed statistical analyses and prepared the manuscript. All authors read and approved the final manuscript.
